# Determination of Natural *In Vivo* Noble-Gas Concentrations in Human Blood

**DOI:** 10.1371/journal.pone.0096972

**Published:** 2014-05-08

**Authors:** Yama Tomonaga, Matthias S. Brennwald, David M. Livingstone, Geneviève Tomonaga, Rolf Kipfer

**Affiliations:** 1 Eawag, Swiss Federal Institute of Aquatic Science and Technology, Department of Water Resources and Drinking Water, Duebendorf, Switzerland; 2 Institute of Biogeochemistry and Pollutant Dynamics, Swiss Federal Institute of Technology (ETH), Zurich, Switzerland; 3 Ergomotion, Losone, Switzerland; 4 Institute of Geochemistry and Petrology, Swiss Federal Institute of Technology (ETH), Zurich, Switzerland; University of Barcelona, Faculty of Biology, Spain

## Abstract

Although the naturally occurring atmospheric noble gases He, Ne, Ar, Kr, and Xe possess great potential as tracers for studying gas exchange in living beings, no direct analytical technique exists for simultaneously determining the absolute concentrations of these noble gases in body fluids *in vivo*. In this study, using human blood as an example, the absolute concentrations of all stable atmospheric noble gases were measured simultaneously by combining and adapting two analytical methods recently developed for geochemical research purposes. The partition coefficients determined between blood and air, and between blood plasma and red blood cells, agree with values from the literature. While the noble-gas concentrations in the plasma agree rather well with the expected solubility equilibrium concentrations for air-saturated water, the red blood cells are characterized by a distinct supersaturation pattern, in which the gas excess increases in proportion to the atomic mass of the noble-gas species, indicating adsorption on to the red blood cells. This study shows that the absolute concentrations of noble gases in body fluids can be easily measured using geochemical techniques that rely only on standard materials and equipment, and for which the underlying concepts are already well established in the field of noble-gas geochemistry.

## Introduction

The atmospheric noble gases He, Ne, Ar, Kr, and Xe are widely used as tracers to analyze the dynamics of aquatic systems such as lakes, oceans, and groundwaters [1], [2]. Recently, new methods have been developed to determine noble-gas concentrations in the porewater of bulk samples of unconsolidated sediment that include both liquid and solid phases (i.e., both porewater and sediment matrix) [3], [4]. These methods have been successfully employed to study the physical transport of fluids in the pore space of lacustrine sediments [5]–[7] and to reconstruct past climate conditions using the concentrations of noble gases dissolved in the sediment porewater [8], [9]. Here we present the results of a preliminary study that might extend the use of these methods to another type of aquatic system: the human body.

In contrast to chemically reactive gases such as O_2_, the partitioning and exchange of noble gases are controlled only by physical processes. This makes noble gases potentially ideal tracers for studying the physical processes controlling gas exchange between (human) body fluids and the ambient atmosphere. For instance, using blood as an example, the rates of gas uptake in the lung can be quantified from mass balances determined from the rebreathing of inert test gases. These mass balances are used to estimate cardiac output by quantifying the flow of blood through the lungs, using models of the circulation of blood within the body and of the transfer of gases from the alveoli of the lung to the blood [10]. However, such rebreathing methods are often observed to underestimate the true cardiac output, possibly because of uncertainties in the underlying mass-balance models [11]. The precise, simultaneous measurement of inert trace gases such as He, Ne, Ar, Kr, and Xe has the potential to overcome the limitations of these models. Physiological studies using noble gases have focused mainly on the heavier elements Kr and Xe because of their anaesthetic properties [12] and because their hyperpolarized isotopes (e.g., ^83^Kr and ^129^Xe) can be used in magnetic resonance imaging (MRI) [13], [14]. Various *in vitro* studies (i.e., gas re-equilibration experiments) with radioactive ^85^Kr [15] and ^133^Xe [15]–[18] have been conducted to determine the solubility of these gases in human blood (Tables 1 and 2). *In vitro* experiments using stable isotopes of He [19], Ne [19], Ar [19], Kr [20], [21], and Xe [20]–[22] yield similar results to those of the *in vitro* experiments using radioactive isotopes (Table 1). The analytical techniques used to determine noble-gas solubilities using stable isotopes are degassing [20], gas chromatography (GC) [19], [21], and gas chromatography combined with mass spectrometry (GC-MS) [22]. *In vivo* studies (i.e., infusion experiments) with the same goal have been conducted using radioactive ^85^Kr [23], [24] and ^133^Xe [24]. The use of such radioactive species allows dissolved-gas concentrations to be determined relatively simply using a scintillation counter, but involves health risks to the test subjects and to the scientific personnel. Further, strict safety regulations apply to the handling of such gases. It should be noted that no measurements have yet been made of the *in vivo* concentrations of noble gases of natural – i.e., atmospheric – origin in human blood, and that all relevant previous experiments have relied on the use of artificial gas tracers.

**Table 1:** Noble-gas partition coefficients between whole blood and air as determined in this study for two subjects (S1 and S2) compared with values from the relevant literature.

	He	Ne	Ar	Kr	Xe
Volume fraction in dry air [26]	(5.24±0.05)·10^−6^	(1.818±0.004)·10^−5^	(9.34±0.01)·10^−3^	(1.14±0.01)·10^−6^	(8.70±0.1)·10^−8^
This study – subject S1	0.0085±0.0004	0.0094±0.0004	0.030±0.001	0.055±0.002	0.120±0.005
This study – subject S2	0.0075±0.0003	0.0081±0.0004	0.026±0.001	0.051±0.002	0.118±0.007
Hardewig *et al.* (1960) [23]				0.058±0.003^a^	
Edwards *et al.* (1963) [19]	0.0085	0.0093	0.0300		
DeBon & Featherstone (1964), cited in [22]					0.20
Isbister *et al.* (1965) [16]					0.142±0.002^b^
Veall & Mallet (1965) [17]					0.146±0.004^b^
Yeh & Peterson (1965) [20]				0.058±0.004^d^	0.181±0.004^c^^,^ d
Muehlbaecher *et al.* (1966) [21]				0.075±0.005	0.149±0.006^c^
Rochester *et al.* (1967) [24]				0.059	0.134^c^
Ladefoged & Andersen (1967) [18]					0.129^c^
Kitani (1972) [15]				0.058^b^	0.136^b^
Hlastala *et al.* (1980) [31]	0.00796±0.00002		0.0305±0.0002		
Goto et al. (1998) [22]					0.115±0.008

The partition coefficients are expressed as nondimensional ratios of the gas concentration in blood (in cm^3^_STP_ of gas per cm^3^ of whole blood, assuming a whole blood density of 1.06 g/cm^3^, where 1 cm^3^_STP_ = 22414^−1^ Mol) to the corresponding volume fraction of the gas in dry air (in vol/vol).

a Based on the linear relationship between hematocrit and partition coefficient in [23].

b Calculated using a linear relationship between the partition coefficients of red blood cells (40%) and plasma (60%).

c Coefficients as calculated in [22].

d Errors represent the standard deviations of the original measurements.

**Table 2:** Noble-gas partition coefficients between blood plasma and red blood cells as determined in this study for one subject (S1) compared with values from the relevant literature.

	He	Ne	Ar	Kr	Xe
This study – subject S1	0.83±0.04	0.81±0.03	0.71±0.03	0.63±0.03	0.51±0.02
Hardewig *et al.* (1960) [23]				0.75±0.04^a^	
Isbister *et al.* (1965) [16]					0.509±0.005
Veall & Mallet (1965) [17]					0.49±0.01
Ladefoged & Andersen (1967) [18]					0.47±0.02
Kitani (1972) [15]				0.691	0.486
Hlastala *et al.* (1980) [31]	1.194±0.008^b^		0.834±0.008^b^		

The partition coefficients are expressed as nondimensional ratios of the gas concentration in the plasma (in cm^3^_STP_ of gas per cm^3^ of plasma, with an assumed plasma density of 1.025 g/cm^3^) to the gas concentration in the red blood cells (in cm^3^_STP_ of gas per cm^3^ of red blood cells, with an assumed red blood cell density of 1.125 g/cm^3^). Note that the values listed are based on the separation of the two phases in a single sample only and cannot therefore be considered as generally representative.

a Measurement error for red blood cells estimated from the maximum deviation from a linear interpolation [23].

b Calculated using a linear relationship between the partition coefficients of red blood cells (44%) and plasma (56%).

Here we suggest tailoring methods developed for the geochemical investigation of the porewater of unconsolidated lacustrine sediments [3], [4] to determine the *in vivo* concentrations of noble gases and the ratios of their isotopes in body fluids, using human blood as an example. In principle, the analytical determination of noble-gas concentrations in blood poses the same experimental challenges as their determination in lacustrine or oceanic sediments. After being extracted from the body, blood starts to coagulate and clusters of coagulated blood enclose a certain volume of the fluid to be analyzed. Complete extraction of all noble gases from these clusters using the standard analytical techniques conceived for bulk water samples [25] is not possible, as the clusters act as a diffusive barrier that might also foster isotope fractionation. Suitably adapted, the two geochemical noble-gas analysis methods mentioned above offer a promising approach to determining noble-gas concentrations in blood. Following one of these methods [4], the separation of plasma and red blood cells by centrifugation should allow the noble-gas concentrations in the plasma phase to be determined, while use of the other method [3] might overcome any problems related to the presence of coagulated blood clusters by the forcible extrusion and expansion of the red blood cell mass into a large extraction vessel, thus allowing the noble gases to be extracted efficiently. Use of these two methods should render the use of anticoagulants (e.g., heparin), which might introduce contamination, unnecessary.

In this work we briefly report on the results of a preliminary study in which these adapted geochemical methods were used to measure noble-gas concentrations in a limited number of samples of human blood. To confirm the accuracy of the methods, for these samples we also present the calculated partition coefficients of the noble gases between whole blood and air, and between blood plasma and red blood cells, and compare the values obtained with values from the literature. Note that our intention here is not to present an extensive analysis of the specific case of the partitioning coefficients of noble gases in human blood: such an analysis would have to be conducted within the context of full clinical trials, and does not lie within the scope of the present methodological paper. Our intention is rather to demonstrate that methods adapted from the field of geochemistry are capable of simultaneously determining the concentrations of all atmospheric noble gases in human body fluids, thus potentially opening up a new research field. The analysis of a few blood samples suffices to demonstrate the method, but does not allow generalizations to be made about the effect on the partitioning coefficients of specific factors such as, for example, blood lipid content. We would encourage physiological researchers to conduct trials using the methods described here to investigate thoroughly the effects of such factors on the noble-gas partitioning coefficients.

The noble-gas composition of Earth’s atmosphere is homogeneous and constant in time and space [26], [27]. This means that when using measurements of the concentrations of naturally occurring atmospheric noble gases dissolved in any liquid–including body fluids–to elucidate physical gas-exchange processes, the concentrations of noble gases in common atmospheric air can be used as stable, well-constrained boundary conditions. Minor changes in the relative composition of atmospheric air, such as a local enrichment with other trace gases such as H_2_, CH_4_, or Rn, have no analytically detectable effect on the partial pressures of the noble gases; under common environmental conditions such changes are therefore orders of magnitude smaller than the analytical uncertainties in measurements made by any noble-gas laboratory. The constancy of composition of the Earth’s atmosphere with respect to noble gases allows the use of artificial (often radioactive) tracers to be avoided, and also means that elemental and isotopic fractionation can be used to identify contamination or gas loss, providing direct confirmation of measurement quality. Because the atmosphere sets well-defined boundary conditions that constrain gas partitioning, the findings set out below also make it possible to relate the concentrations of the atmospheric noble gases directly to the concentrations of other atmospheric gases such as N_2_ and O_2_.

## Materials and Methods

The two methods employed here for noble-gas analysis are described in detail in the relevant geochemical publications [3], [4]. These methods were adapted to measure the noble-gas concentrations in blood as described in the following paragraphs. Both noble-gas quantification methods use the same noble-gas purification line [25]. The analytical techniques employed are well established in the field of noble-gas geochemistry and allow highly precise and accurate determination of noble-gas concentrations. Both methods employ standard materials and devices available from commercial suppliers of vacuum and medical equipment.

**Figure 1:**
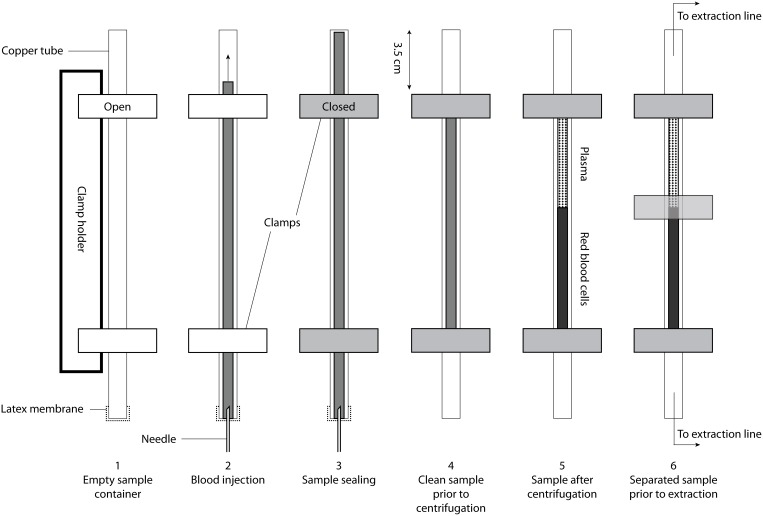
Acquisition and preparation of a blood sample for noble-gas analysis. Whole blood in injected from a syringe through a hypodermic needle into a copper tube held vertically (Steps 1–2). After closing the sample with two special metal clamps (Step 3), the copper tube is cleaned (Step 4) and centrifuged at 2300 rpm in a centrifuge with a swing-out rotor (Step 5). After centrifugation, the plasma and red blood cells are separated using a third clamp (Step 6).

Venous blood was taken from two of the authors of this manuscript (one sample from subject S1 and two samples from subject S2). The sample from subject S1 was taken using a 10-mL hypodermic syringe and transferred immediately to a copper tube that was used as a sampling container. To avoid contamination, particular care was taken to prevent the occurrence of air bubbles in the syringe and in the copper tube. To this end, the copper tube was held vertically, its bottom end was sealed with a latex membrane, and the blood (with a total volume of about 10 mL) was injected slowly through the membrane into the copper tube (Fig. 1). During this process, only the tip of the syringe needle (∼5 mm) penetrated through the latex membrane. In the case of subject S2, the two samples were transferred into the two vertical copper tubes directly using a cannula. In one of the samples from subject S2 a small amount of heparin was injected into the copper tube prior to sampling to inhibit coagulation. In all cases the flow of blood into the copper tube was intentionally slow, ensuring that the bottom part of each copper tube was filled completely with blood so that no air bubbles were trapped in the blood mass. Such a vertical setup limits gas exchange with the atmosphere across the surface of the rising blood meniscus within the copper tube. Immediately after the blood was transferred into the copper tube, the latter was sealed air-tight at both ends by pinching them off with two special metal clamps [4]. The clamps were set at a distance of about 3.5 cm from the openings of the copper tube to ensure that even diffusive gas exchange could not affect the sample. (These two short segments of empty copper tube also functioned as connectors to the noble-gas purification line.).

In accordance with the preparation procedure [4], the sample from subject S1 was centrifuged for about 2 hours in a Heraeus UJ3S centrifuge at 2300 rpm (corresponding to a radial acceleration of about 2.3·10^4 ^m/s^2^) at an ambient temperature of approximately 20°C to separate the blood plasma from the red blood cells within the closed copper tube. After centrifugation a third metal clamp was set on the copper tube between the plasma and the red blood cells [4]. This third clamp was located in the middle of the copper tube to achieve a good separation of the two phases (based on a normal hematocrit of ∼48% for men [28]). In future experiments we would recommend determining the geometrical position of the interface between the plasma and the red blood cells empirically (e.g., by centrifuging an additional blood aliquot in a glass vial with the same dimensions as the copper tube).

Noble-gas concentration determinations and isotopic analyses were carried out in the Noble Gas Laboratory of the Swiss Federal Institute of Science and Technology in Zurich. For the sample from subject S1, the noble-gas concentrations in the part of the copper tube containing the plasma were measured according to the original analytical protocol [4], and the noble-gas concentrations in the part containing the red blood cells were determined using the extrusion method [3]. The latter experimental protocol involves heating the samples up to 150°C and forcibly extruding them into an extraction vessel under ultra high vacuum (UHV) conditions. A wad of silver wool inserted into the outlet of the extraction vessel prevented blood clusters from entering and contaminating the purification line. After extruding the red blood cells from the copper tube into the extraction vessel, the latter was heated homogeneously for about 30 minutes (heating fosters the release of dissolved noble gases from the blood mass into the gas phase). The pressure within the extraction vessel was kept below 330 hPa by regulating the temperature of the extraction vessel, while the temperature of the copper tube was kept constant at 250°C. Before the gases were allowed to enter the purification line, the extraction vessel was cooled down to ambient air temperature and the copper tube to a constant 50°C. During extraction of the noble gases from the plasma, the extraction vessel, along with the attached copper tube, was shaken to facilitate the release of gases from the liquid phase. After purification, the noble-gas concentrations and the isotope ratios were determined in two static mass spectrometers according to well-established experimental protocols commonly used to determine noble-gas concentrations in water samples [25], [29], [30]. No further details on the purification line are given as such details depend crucially on the specific analytical setup of each noble-gas laboratory.

The heparinized whole blood sample from subject S2 was analyzed following the same protocol as that for the plasma phase of the sample from subject S1 [3]. The noble-gas concentrations in the non-heparinized whole blood sample from subject S2 were measured according to the extrusion method used for the red blood cells of the sample from subject S1 [4]. The samples from subject S2 were intended to supply additional data to improve our assessment of the general applicability of the new methods.

The error in a single measurement is given by the uncertainty associated with the regression algorithm applied to compensate for ion-pumping or memory effects during the analysis. For a measured amount of gas, the total error corresponds to the standard deviation of the calibration signals combined with the error in the single measurement. The combination of slow calibrations (i.e., well-defined aliquots of atmospheric air that set the basis for determining absolute noble-gas concentrations) and fast calibrations (i.e., gas aliquots from calibration bottles with a known noble-gas composition differing from that of atmospheric air) allows the fluctuations in sensitivity of the mass spectrometers to be assessed and corrected on a daily basis. For common water samples, typical precisions for the concentration measurements are ±0.8% (He), ±0.9% (Ne), ±0.3% (Ar), ±0.8% (Kr), and ±1.0% (Xe). For the isotope ratios, typical precisions are ±0.7% (^3^He/^4^He), ±0.3% (^20^Ne/^22^Ne), and ±0.2% (^36^Ar/^40^Ar) [25]. The larger uncertainties in the measurements presented in this work, which are similar to those affecting the determination of noble-gas concentrations in the porewater of unconsolidated lake sediments [3], [4], result mainly from the one order of magnitude smaller sample volume (i.e., ∼5 mL of blood as opposed to a common sample size of 45 mL for water).

Each noble-gas analysis required a (sub)sample volume of only ∼5 mL. Previous studies using GC [19] or GC-MS [22] techniques that required a similarly small amount of blood depended on the equilibration experiments being conducted on enriched gas phases (30–100% noble gases), which increased the concentrations of noble gases in the sample by several orders of magnitude with respect to the natural concentrations in blood. The overall sensitivity of the two methods adopted in the current study should allow the natural noble-gas concentrations in (human) blood to be determined based on a subsample volume of as little as 1–2 mL.

### Ethics Statement

This study was conducted according to the principles expressed in the Declaration of Helsinki. The results presented here are based on self-experiments, in which two of the authors, without external assistance, acquired a total of three samples of their own blood for analytical purposes only. The directorate of Eawag gave written authorization for this study. According to the guidelines of the Ethics Commission of the Canton of Zurich (Switzerland), the study requires neither participant consent nor a statement from the cantonal ethics committee (Swiss Federal Acts 812.21 and 813.21).

## Results and Discussion

### Reproducibility

**Figure 2:**
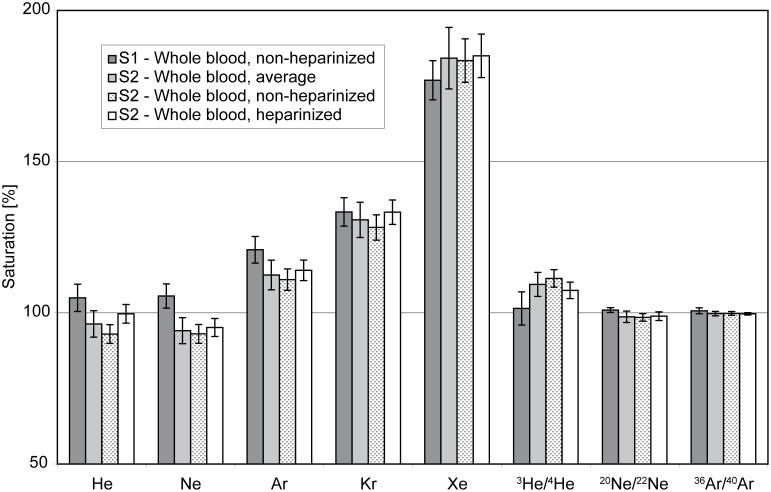
Percentage saturation of whole human blood from subjects S1 and S2 with various noble gases and noble-gas isotope ratios under different environmental conditions (see Table 3 for details). The agreement between the measurements conducted on the two subjects demonstrates that the methods yield reproducible results.

The whole-blood noble-gas concentrations and isotope ratios reported in Table 3 and displayed in Fig. 2 as saturations normalized to the expected concentrations for air-saturated water (ASW) allow straightforward assessment of the reproducibility of the measurements. The solubility functions used to calculate the ASW concentrations of noble gases in saline solutions are generally based on empirical solubility data for seawater and NaCl solutions. Such solubility parameterizations allow noble-gas equilibrium concentrations to be calculated as a function of pressure, salinity, and temperature. This study makes use of the noble-gas solubility functions recommended for aquatic systems [1].

**Table 3:** Noble-gas concentrations, noble-gas isotope ratios and standard errors (at the 1σ level) measured in blood samples from two subjects (S1 and S2). ASW (S1)  =  noble-gas concentrations in air-saturated water at a temperature of 36.5°C, a salinity of 9 g/kg and an altitude of 225 m above sea level (corresponding to an atmospheric pressure of 986.3 hPa).

	Concentrations (cm^3^STP/g)					Isotope ratios		
	He (10^−8^)	Ne (10^−7^)	Ar (10^−4^)	Kr (10^−7^)	Xe (10^−9^)	^3^He/^4^He (10^−6^)	^20^Ne/^22^Ne	^36^Ar/^40^Ar (10^−3^)
ASW (S1)	3.99	1.53	2.15	4.43	5.57	1.38	9.80	3.38
ASW (S2)	3.85	1.47	2.05	4.21	5.26	1.38	9.80	3.38
S1– Whole blood	4.2±0.2	1.62±0.06	2.60±0.09	5.9±0.2	9.9±0.4	1.40±0.08	9.89±0.08	3.41±0.03
S1– Blood plasma	4.0±0.2	1.52±0.06	2.28±0.08	4.8±0.2	7.1±0.3	1.43±0.07	10.03±0.08	3.42±0.03
S1– Red blood cells	4.39±0.07	1.72±0.03	2.92±0.04	7.0±0.1	12.6±0.2	1.37±0.04	9.75±0.01	3.40±0.01
S2– Whole blood average	3.7±0.2	1.38±0.06	2.3±0.1	5.5±0.2	9.7±0.5	1.51±0.05	9.7±0.2	3.37±0.02
S2– Whole blood non-heparinized	3.6±0.1	1.37±0.05	2.27±0.07	5.4±0.2	9.7±0.4	1.54±0.04	9.7±0.1	3.38±0.02
S2– Whole blood heparinized	3.8±0.1	1.40±0.04	2.34±0.07	5.6±0.2	9.7±0.4	1.49±0.04	9.7±0.1	3.37±0.01

ASW (S2)  =  noble-gas concentrations in air-saturated water at a temperature of 37.5°C, a salinity of 9 g/kg and an altitude of 490 m above sea level (corresponding to an atmospheric pressure of 955.5 hPa). STP  =  standard temperature (0°C) and pressure (1 atm = 1013.25 hPa).

ASW concentrations were calculated for the local atmospheric pressure at each sampling position, the subjects’ body temperature, a blood-like salinity of 9 g/kg, and the individual partial pressures of water vapor at saturation (for more details refer to Table 3). In general, all measured noble-gas concentrations and isotope ratios agree well with each other, indicating that the methods described here yield reproducible measurements. Note, however, that the noble-gas measurements are of course valid only for each subject at the time and location the relevant sample was taken, and should not be considered generally representative, as the dependence of these measurements on biological variables such as lipid content and hematocrit cannot be assessed.

The small deviations between S1 and S2 observed for Ne can be explained by the statistics of the 1σ measurement errors, as ∼30% of the measurements would be expected to lie outside the 1σ confidence interval. The same applies to the He concentrations of the non-heparinized and the heparinized samples of subject S2. Despite the fact that the difference in the He concentrations in the two S2 samples is not statistically relevant within the context of the present comparison, the higher He concentrations in the heparinized sample might indicate a slight contamination resulting from the dissolution of air trapped in the heparin pellets added to the blood sample (analogous to the formation of excess air in the ocean or in groundwater by the interaction between air bubbles and water [1]). The use of heparin-coated instruments would remove this potential source of contamination.

The agreement of the noble-gas concentrations and isotope ratios in the whole blood samples from two different subjects taken under different environmental conditions underlines the robustness of the concepts of physical gas partitioning between gaseous and liquid phases that underlie this study [1], [26]. The data demonstrate that the atmosphere sets very well defined boundary conditions that can be inferred for each subject using empirical solubility equations.

### Elemental Concentration Patterns

**Figure 3:**
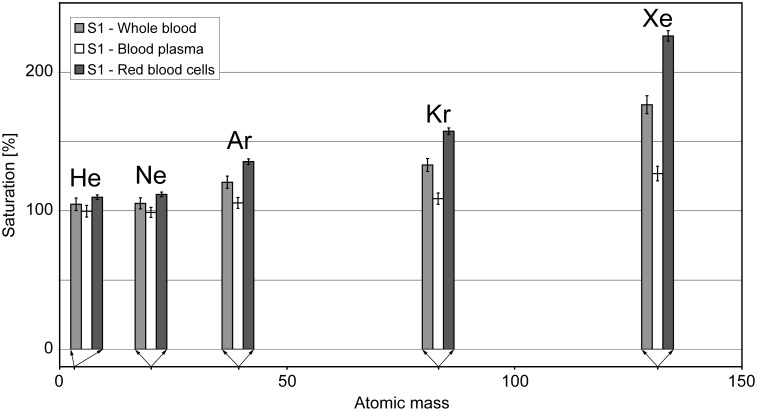
Percentage saturation of human blood plasma (white), red blood cells (black) and whole blood (gray) with noble gases relative to air-saturated water (at a temperature of 36.5°C and a salinity of 9 g/kg at an altitude of 225 m a.s.l.) measured in the sample from subject S1. For red blood cells, the saturation increases with the atomic mass of the noble gas.

The noble-gas concentrations in the plasma of the sample from subject S1 (Table 3) are generally close to those found in ASW. The only evident exception to this is Xe, which shows a supersaturation of ∼30% with respect to ASW, possibly resulting from the presence of some erythrocytes in the plasma (see below). By contrast, the percentage saturation of the noble gases in the red blood cells follows a distinct pattern, increasing with atomic mass (Fig. 3). This observation agrees with the results of previous studies on noble-gas solubility in human blood [15]–[24], [31].

### Noble-gas Isotope Ratios

The ^3^He/^4^He, ^20^Ne/^22^Ne, and ^36^Ar/^40^Ar isotope ratios in the whole-blood samples (plasma plus red blood cells in the case of subject S1) are all close to the theoretical values for ASW (Table 3 and Fig. 1), reflecting the atmospheric origin of the dissolved gases. Nevertheless, based on the 1σ measurement errors given in Table 3, the ^20^Ne/^22^Ne isotope ratio in the whole-blood sample from subject S1 is significantly higher than the expected ASW value. Looking separately at the isotope ratios in the blood plasma and the red blood cells, there is no significant difference between the two in the case of ^3^He/^4^He, but the ^20^Ne/^22^Ne and ^36^Ar/^40^Ar isotope ratios are significantly higher in the blood plasma than in the red blood cells. All isotope ratios measured in the red blood cells are closer to the respective ASW values than are the isotope ratios measured in the plasma (which in all cases are higher than the ASW values). In the case of ^20^Ne/^22^Ne, the significant deviation between the isotope ratio in the whole-blood sample and the expected ASW value thus originates from the plasma and not from the red blood cells. By contrast, the ^36^Ar/^40^Ar isotope ratio in the whole-blood sample would seem to be affected also by a slight enrichment of the red blood cells with the lighter isotope. The fact that the values of ^20^Ne/^22^Ne and ^36^Ar/^40^Ar measured in the plasma deviate from the expected values for ASW suggests that fractionation processes are likely occurring. Although the ^3^He/^4^He isotope ratios in the whole-blood samples from subjects S1 and S2 are essentially identical, the consistently higher ^3^He/^4^He values in the samples from subject S2 (being affected by smaller errors) suggest that the He isotope ratio might also be affected by such fractionation processes. However, because there are no previous studies reporting noble-gas isotope ratios in human blood, the paucity of available data means that any further interpretation would be merely speculative.

### Partition Coefficients between Whole Blood and Air

In general, the partition coefficients between whole blood and air (Table 1) are close to the range of values found in the literature. The partition coefficients for He (S1: 0.0085±0.0004; S2: 0.0075±0.0003), Ne (S1: 0.0094±0.0004; S2: 0.0081±0.0004), and Ar (S1: 0.030±0.001; S2: 0.026±0.001) are in especially close agreement with the results of previous studies [19], [31]. The partition coefficients found for Kr (S1: 0.055±0.002; S2: 0.051±0.002), even though in good agreement with previously reported values [15], [20], [23], [24], seem to be generally lower than expected (Table 1). The partition coefficients obtained for Xe (S1: 0.120±0.005; S2: 0.118±0.007) are significantly lower than values reported in the older literature [15]–[18], [20], [21], [24], but agree within the range of error with a more recently determined value (0.115±0.008) (where in this case ±0.008 is the 95% confidence interval) [22]. Based on their measurements, the authors of the more recent study hypothesized that the blood/air partition coefficient for Xe may be lower than the previously generally accepted value [22]. Our measurements support this hypothesis and, in particular when considering the results for subject S2, suggest that the same can be postulated for the partition coefficients of Ar and Kr.

### Partition Coefficients between Plasma and Red Blood Cells

The partition coefficients between the blood plasma and red blood cells for the sample from subject S1 are presented in Table 2. As the presence of minimal amounts of red blood cells in the plasma cannot be excluded, a slight bias in these partition coefficients is possible, especially since they are based on a single sample. Nevertheless, the values obtained are similar to those in the literature (Table 2), indicating that separation of the two phases within the sealed copper tube was successfully achieved. For Xe, for instance, the partition coefficient obtained in this study (0.51±0.02) agrees rather well with the ones obtained in four previous studies [15]–[18].

Precise determination of the hematocrit turns out to be very useful in achieving complete separation of plasma and red blood cells. Hence, in future (possibly clinical) studies, the hematocrit should preferably be measured rather than estimated, since it is affected by the centrifugation time and the acceleration experienced by the samples. By enabling the achievement of complete (or at least a well-defined) separation of plasma and red blood cells, measurement of the hematocrit would also likely allow the noble-gas concentrations in the “pure” red blood cells to be determined more precisely. The red blood cells are expected to trap a mass of plasma corresponding to ∼3.5% of the packed cell volume (or more in the case of non-heparinized blood because of the formation of a fibrin net).

For subject S1 we explicitly avoided using an anti-coagulant such as heparin because of its potential as a source of contamination. This is likely to result in a lack of clotting proteins in the plasma phase, making it more serum-like. However, the general agreement of our results with the outcome of previous experiments indicates that clotting factors seem not to have a significant effect on noble-gas solubilities.

## Conclusions

This study shows that it is possible to apply simple and robust analytical methods already developed in geochemical studies [3], [4] to determine simultaneously all stable, natural (i.e., atmospheric) noble-gas concentrations and isotope ratios *in vivo* in small subsamples (∼5 mL) of human body fluids without the use of any artificial gas mixture. The accuracy of the determinations is confirmed for the example of human blood by calculating the partition coefficients between the whole blood and atmospheric air, and between the blood plasma and the red blood cells. The results generally agree well with values from the relevant literature. However, the blood/air partition coefficients for Ar, Kr, and Xe determined in this study are somewhat lower than the corresponding partition coefficients obtained in most older studies. This suggests, in agreement with one of the most recent previous studies [22], that the partition coefficients for the heavier noble gases might possibly be lower than previously generally accepted values based on older determinations. However, as the methodological work described here was limited to only a few blood samples, its quantitative results should not be interpreted as having general physiological implications. Instead, this preliminary methodological study should be viewed as an attempt to make inroads into a potentially new field of research in an area of overlap between two fields – geochemistry and physiology – that traditionally have little contact and might be supposed to have very little in common. However, it is at the borders between traditional academic disciplines where cross-fertilization – especially with regard to the development and application of new methods – often gives rise to unexpected advances that can benefit research in both fields.

Future physiological studies based on the methods used here would be useful to investigate the origin of the elemental and isotopic fractionation observed in this work. Further studies could focus on experiments with artificial gas mixtures enriched with stable noble-gas isotopes to better quantify physical gas exchange processes in living beings. In addition, our method may prove useful in studies on the inhalation of Xe or other noble gases with the aim of improving the physical performance of athletes, for instance by stimulating the production of erythropoietin (EPO).

Recent advances in membrane inlet mass spectrometry (MIMS) might allow the online determination of gas breakthrough curves [32], thus avoiding the use of chemically reactive or radioactive gas species. In particular, Ar, which has similar physical properties to O_2_, might be a helpful proxy for investigating the efficiency of pulmonary gas exchange. The data from such studies could provide an experimental basis for the rigorous physical analysis of gas/body fluid partitioning in human tissues based on theoretical concepts of gas/water partitioning in porous media that have already been developed in a geochemical context.
